# How we do it: a method of neck dissection for histopathological analysis

**DOI:** 10.1186/1471-2482-7-21

**Published:** 2007-10-31

**Authors:** Tahwinder Upile, Waseem Jerjes, Seyed Ahmad Reza Nouraei, Sandeep Singh, Peter Clarke, Peter Rhys-Evans, Colin Hopper, David Howard, Anthony Wright, Holger Sudhoff, Cyril Fisher, Ann Sandison

**Affiliations:** 1 The Ear Institute, University College London, London, UK; 2 Department of Head & Neck Surgery, Charing Cross Hospital, UK; 3 Department of Head & Neck Surgery, The Professorial Unit, The Royal National Throat, Nose and Ear Hospital, London, UK; 4 Oral & Maxillofacial Surgery/Head & Neck Unit, University College London Hospital, London, UK; 5 Department of Surgery, Royal Free & University College Medical School, London, UK; 6 Unit of Oral & Maxillofacial Surgery, Division of Maxillofacial, Diagnostic, Medical and Surgical Sciences, UCL Eastman Dental Institute, London, UK; 7 Department of Head & Neck Surgery, The Royal Marsden Hospital, London, UK; 8 Department of Otorhinolaryngology, University of Bochum, Germany; 9 Department of Histopathology, The Royal Marsden Hospital, London, UK; 10 Department of Pathology, Charing Cross Hospital, London, UK

## Abstract

**Background:**

Dissection of the lymphatic structures in the neck is an integral part of the management of many head and neck cancers.

We describe a technique of surgical dissection, preparing the tissue for more precise histological analysis while also reducing operative time and complexity.

**Methods:**

When dissected, each level is excised between lymph nodes groups and put into a separate pot of formalin taking care to avoid rupture of any obvious pathological nodes.

**Results:**

This makes for a simpler dissection as the surgeon progresses, as a larger more cumbersome specimen is avoided and manipulation of involved nodes is actually reduced with a reduced risk of tumour spillage.

**Conclusion:**

We feel that our technique provides several advantages for the histopathologist as well as the surgeon. As the dissection of the specimen into the relevant levels has already been performed, time is saved in orientating and then dissecting the specimen. Accuracy of dissection is also improved and each piece of tissue is a more manageable size for processing and analysis.

This technique may also have several surgical advantages when compared with the commonly practiced techniques e.g. with reducing in-vivo specimen manipulation, hence reducing the risk of inadvertent injury to important structures and tumour spillage.

## Background

A neck dissection specimen when removed "en bloc" (radical neck dissection) and preserved in formalin may be unrecognisable when processed by the histopathology laboratory. Even when labelled or pinned on cork board (orientation plate), the boundaries of the levels of lymph nodes within the neck are surgical boundaries not easy to delineated in the resected specimen. Considering that up to 30% formalin-related post-excision shrinkage may occur, any labelling technique has to be able to withstand the vagaries of processing.

This is more so with the increasing use of the modified radical (selective) neck dissection were preservation of the internal jugular vein and the sternocleidomastiod (SCM) and functional neck dissection further increase the difficulty of orientating the specimen.

Correct and accurate analysis of the lymph nodes surgically excised in a head and neck cancer case is extremely important for the prognostic, diagnostic and treatment information it provides. In the situation of an occult primary, the level of lymph node metastasis will give a clue to the site of origin of disease. Also as increasing levels or more distant levels are affected by disease, so prognosis worsens [1,2].

In a study involving 20 cadaver neck dissections, a quantification of lymph nodes in selective neck dissection took place. The average number of lymph nodes removed for levels I–V was 24, with 13 for levels I–III and 19 for levels II–IV, when compared to other clinical reviews; Friedman et al. concluded that the number of lymph nodes removed in selective neck dissection should be comparable to that of the corresponding levels in radical neck dissection, provided that strict adherence to surgical boundaries is maintained [3].

We describe a technique of level-specific neck dissection in which each level of lymph nodes are dissected separately, and sent for histological analysis separately. This improves the accuracy of the resection as the boundaries of the levels are seen and used at surgery and not arbitrarily taken in the lab. It also shortens the resection time.

## Methods and Results

**Figure 1:**
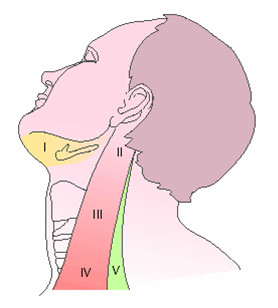
Surgical anatomical boundaries of neck node levels.

The patient is prepared under general anaesthesia in the supine position with the neck extended and turned to the opposite side. We utilise the modified McFee [4] incision as it provides a more acceptable cosmetic scar and avoids the trifurcation scar, which in the situation of wound breakdown risks an exposed carotid artery. Sub-platysmal flaps are raised taking care to preserve the greater auricular and marginal mandibular nerves. We have no standard order of dissection other than as a rule we will dissect the level containing the enlarged lymph nodes early to assess operability and necessitate frozen section analysis if indicated. If structures are fixed the dissection is carried out from an area of normality towards disease. We try to ensure dissection is carried out within cervical fascial compartments retaining the barriers to spread and easing dissection. The boundaries of cervical lymph node levels are surgical anatomical boundaries found at operation are shown in Table 1, also see Figure 1.

**Table 1:** Surgical Levels of the head & Neck

***Level I***	Lower border of the body of the mandible superiorly, posterior belly of the diagastric muscle posteriorly, hyoid bone inferiorly and the midline medially
***Level II***	Base of skull superiorly, lateral limit of the sternohyoid muscle anteriorly, posterior border of the sternocleidomastoid muscle posteriorly, and level of the hyoid bone inferiorly
***Level III***	Level of the hyoid bone superiorly, lateral limit of the sternohyoid muscle anteriorly, the posterior border of sternocleidomastoid muscle posteriorly, and the omohyoid tendon inferiorly
***Level IV***	Omohyoid tendon superiorly, lateral limit of the sternohyoid muscle anteriorly, posterior border of the sternocleidomastoid muscle posteriorly, and clavicle inferiorly
***Level V***	Posterior border of sternocleidomastoid muscle anteriorly, anterior border of the trapezius muscle posteriorly, and clavicle inferiorly

**Figure 2:**
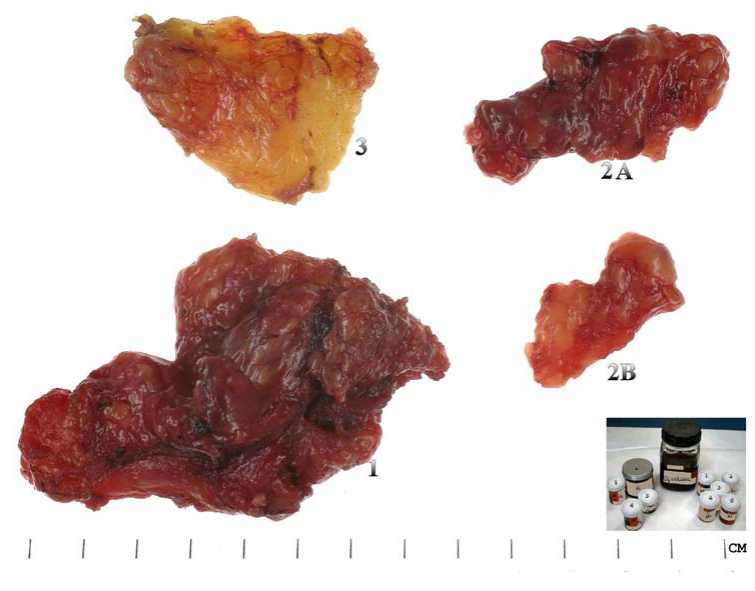
Showing separated left supra-omohyoid neck dissection specimen per level, (left levels 1, 2A, 2B and 3). The inset shows the separate pathology pots for each neck level and each side in addition to the main specimen.

**Figure 3:**
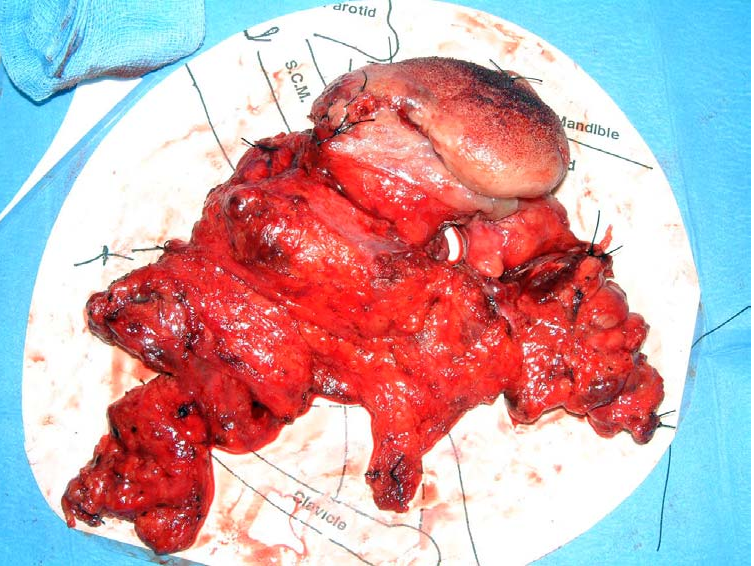
A Modified radical neck dissection specimen. This is a typical example of our previous method of 'en-bloc' resections whereby the tongue/floor of mouth and neck are taken in continuity. The resection has been secured onto a standard neck landmark diagram. Although appearing impressive and attempting to 'help' the pathologist much important data is lost. The bulkiness of the tumour in three dimensions seems to overspill in areas. Also manipulation of the entire specimen during each stage of the excision may easily have shed potential viable tumour cells. Taken objectively and in light of modern molecular biological knowledge many areas of potentially positive resection margins **have not **been sampled. The specimen in contact with areas of concern e.g. mandible, deep resection margins should have be stained.

When dissected each level is excised between lymph nodes groups (sutures may be used to indicate superior and anterior, occasionally ink is used to mark areas of particular interest) and put into a separate pot of formalin taking care to avoid rupture of any obvious pathological nodes (Figures 2). This makes for an easier dissection as the surgeon progresses, as a larger more cumbersome specimen (Figure 3) is avoided and manipulation of involved nodes is actually reduced with a reduced risk of tumour spillage. We perform this type of dissection as a part of our routine clinical practice and have done so for the past 5 years, we have analysed over 200 neck dissection specimens in this time. The neck dissection specimen is divided in theatre by surgeons into the appropriate levels and the seperate pieces are placed in labelled pots containing 10% buffered formalin. This method is more accurate due to the initial division of the tissue by the surgeon and the lymph node yield is higher, allowing more nodes to be assessed for metastases.

In the laboratory the Pathologist cuts the tissue from each pot into pieces that will fit into the cassettes used routinely for histological processing into paraffin wax blocks. Often one tissue piece is bisected and processed in two blocks. This means one lymph node may cut in half and each half processed in a separate block. The block details are recorded so that one lymph node is not counted twice. Individual lymph nodes are not separated out from surrounding connective tissue but are identified at microscopy. Usually all of the tissue is processed from levels 3–5 however, salivary gland tissue often present in levels 1 & 2 tissue may be dissected and sampled and not all processed. Fibro-fatty tissue which often contains lymph nodes is all processed where possible. Sections 3–4 microns thick are cut from the paraffin blocks and usually one section per tissue block processed on a slide. Where the tissue blocks are small, more that one section may be present on a slide. Serial sections at different levels through the block are not routinely requested as this workload would not be sustainable in the routine laboratory. The sections are stained with haematoxylin and eosin. Immunostains for cytokeratin to detect micrometastases are not routinely requested.

## Discussion

The surgeon's responsibility does not end with just taking the excision specimen. A council of perfection would suggest that even before surgery a sampling strategy would be discussed with the pathologist including the location and possibility of any difficult areas and likely positive margins. The caveat is that further information may become available at surgery that may modify this strategy. Thus allowing adequate and appropriate resource allocation e.g. frozen section facility...etc.

In mitigation of a selective neck dissection, Muzaffar K conducted a retrospective, 25-year, study involving patients with untreated head and neck cancer who had squamous cell carcinoma (SCC) metastatic to cervical lymph nodes on histological examination and were treated with a selective, modified, or radical neck dissection. Evidence of recurrence was 3.3% in the selective neck dissection group and 5.2% in the radical and modified neck dissection group. Disease-free (2-year survival) was 80% in the selective neck dissection group and 64% in the radical and modified neck dissection group [5].

However, advanced nodal involvement can sometimes justify a more aggressive resection technique. Schiff et al. concluded that a selective neck dissection may be sufficient for many N+ patients with SCC, but some patients with extensive nodal disease may benefit from more aggressive treatment of the neck [6].

Jaehne et al. evaluated whether intra-operative macroscopic inspection of the sternocleidomastoid muscle in regard to tumour infiltration is sufficient to decide about muscle resection and whether there are prognostic differences between patients undergoing radical versus modified radical (selective) neck dissection. In a study involving 337 patients, they concluded that intraoperative inspection of the SCM constitutes a valid parameter for deciding whether tumour infiltration is present or not and there were no statistically significant prognostic differences (2-year, 5-year and 10-year-survival) between stage III and IV patients with oral cavity, oropharyngeal, hypopharyngeal and laryngeal carcinomas treated by either radical or selective neck dissection [7].

We have already discussed the erroneous assumption of the superiority of the naked eye "en-bloc" dissection over "selective" dissection between the nodes groups without node rupture [2]. This is due in part upon the "en-bloc" enthusiasts mis-appreciation of true microscopic disease spread (via microlymphatics) and the tendency for many resections to reflect surgical ease or expediance despite their scope or duration [2]. By answering this basic criticism of "selective" neck dissection we logically extend this rationale to histopathological sampling.

The problem we aim to resolve is that of perioperative co-registration of pathology with anatomy. Unfortunately, the classification of cervical lymph node levels also differ from those suggested by pre-operative radiological imaging, however this accuracy is related to neck level being imaged. This also has important implications for the anatomico-pathological accuracy of radiologically directed biopsy (Fine Needle Aspiration and if oncologically justified core biopsy). Since this may lead to errors in the pre-operative planning of the correct selective neck dissection carried out by the surgeon with consequent increase in locoregional recurrence or residual disease.

The presence of cervical nodal metastatses in head and neck cancer and the increasing number of levels involved worsens the prognosis [1]. Determining the degree of nodal spread is important in firstly eradicating the disease and secondly in the decision regarding the use of postoperative treatment e.g. radiotherapy. With new techniques of delivery of radiotherapy such as intensity modulated radiotherapy [8], in which a radical treatment dose can be selectively applied to the involved nodal level, uninvolved levels and normal tissues can be spared reducing the associated morbidity of radical radiotherapy. It is imperative that there is accurate and reproducible co-registration of disease and *in situ *lymph node level. This is important considering the surgically directed brachytherapy treatment range (~1 cm) which spares more distant tissues from radiation toxicity. This ensures better dose delivery and may account for some of the variations in survival rates.

Bhattacharyya N found that the modified radical neck dissection and functional neck dissection, when compared with radical neck dissection, do not compromise the quantity of cervical nodes excised [9].

Accurate histological analysis of the resected specimen is therefore mandatory in managing these patients. Labelling techniques of neck dissection specimens have been described previously [10,11], which can aid the histo-pathologist. However, we feel that our technique provides several advantages for the histo-pathologist as well as the surgeon. As the dissection of the specimen into the relevant levels has already been performed, time is saved in orientating and then dissecting the specimen. Accuracy of dissection is also improved and each piece of tissue is a more manageable size for processing and analysis.

## Conclusion

This technique may have several surgical advantages when compared with the commonly practiced techniques. In a comprehensive neck dissection in which preservation of both the accessory nerve and internal jugular vein are goals, both of these structures are skeletonised and in doing this with a bulky cumbersome specimen which needs constant manipulation, there is increased risk of inadvertent injury to these structures and tumour spillage.

Furthermore, as illustrated in Figure 3 the actual three-dimensional bulk of the tumour cannot be encompassed by the standard two dimensional anatomical template upon which the neck dissection is secured. It is again the responsibility of the operating surgeon to take the knife and define the exact surgical borders of neck dissection levels rather than merely indicating a proxy.

## Competing interests

The author(s) declare that they have no competing interests.

## Authors' contributions

TU, WJ, SN, SS, HS, CF, AS: designed the study, carried out the literature research, clinical study and manuscript preparation.

PC, PR, CH, DH, AW: contributed to conception and design, carried out the manuscript editing and manuscript review.

All authors read and approved the final manuscript.

## Pre-publication history

The pre-publication history for this paper can be accessed here:


